# Spontaneous quorum-sensing hierarchy reprogramming in *Pseudomonas aeruginosa* laboratory strain PAO1

**DOI:** 10.1186/s13568-022-01344-7

**Published:** 2022-01-26

**Authors:** Xiaoyan Cheng, Mingqi Lu, Huifang Qiu, Yuanhao Li, Linfeng Huang, Weijun Dai

**Affiliations:** 1 Guangdong Laboratory for Lingnan Modern Agriculture, South China Agricultural University, Guangzhou, 510642 China; 2 Guangdong Province Key Laboratory of Microbial Signals and Disease Control, Integrative Microbiology Research Center, South China Agricultural University, Guangzhou, 510642 China; 3 Department of Biomedical Sciences, City University of Hong Kong, Kowloon, Hong Kong SAR China; 4 Division of Natural and Applied Sciences, Duke Kunshan University, Kunshan, Jiangsu China

**Keywords:** Quorum sensing, LasR, MexT, *Pseudomonas aeruginosa*, Bacterial communication

## Abstract

*Pseudomonas aeruginosa* strain PAO1 has been commonly used in the laboratory, with frequent genome variations reported. Quorum sensing (QS), a cell–cell communication system, plays important role in controlling a variety of virulence factors. However, the evolution and adaptability of QS in those laboratory strains are still poorly understood. Here we used the QS reporter and whole-genome sequencing (WGS) to systematically investigate the QS phenotypes and corresponding genetic basis in collected laboratory PAO1 strains. We found that the PAO1-z strain has an inactive LasR protein, while bearing an active Rhl QS system and exhibiting QS-controlled protease-positive activity. Our study revealed that an 18-bp insertion in *mexT* gene gave rise to the active QS system in the PAO1-z strain. This MexT inactivation restored the QS activity caused by the inactive LasR, showing elevated production of pyocyanin, cyanide and elastase. Our results implied the evolutionary trajectory for the PAO1-z strain, with the evulutionary order from the first Las QS inactivation to the final Rhl QS activation. Our findings point out that QS homeostasis occurs in the laboratory *P. aeruginosa* strain, offering a potential platform for the QS study in clinical isolates.

**Supplementary Information:**

The online version contains supplementary material available at 10.1186/s13568-022-01344-7.

## Introduction

*Pseudomonas aeruginosa* is an opportunistic pathogen that causes several severe acute and chronic human infections, including the infections in cystic fibrosis (CF) patients with compromised immune systems (Gellatly and Hancock 2013; Klockgether and Tümmler 2017). A variety of *P. aeruginosa* virulence factors are regulated by the quorum-sensing (QS) system (Papenfort and Bassler 2016). QS is a bacterial cell–cell communication system that controls the expressions of hundreds of genes in *P. aeruginosa*. Two acyl-homoserine lactone (AHL) QS systems, LasI-LasR and RhlI-RhlR, were identified in *P. aeruginosa*. LasI and RhlI catalyze the productions of diffusible QS signal N-3-oxododecanoyl homoserine lactone (3OC12-HSL) and butyryl-HSL (C4-HSL), respectively. 3OC12-HSL-bound LasR activates the expression of downstream genes. C4-HSL-bound RhlR acts analogously to 3OC12-HSL-bound LasR. The activation of Rhl QS system requires the induction of the Las QS system. These two AHL QS systems also interact with a Pseudomonas Quinolone Signal (PQS) system. In general, the LasI-LasR is atop the QS hierarchy and deletion of LasI or LasR results in the inactivation of the whole QS system (Lee and Zhang 2015).

MexT is a transcriptional regulator of the LysR family and positively controls the MexEF-OprN efflux pump (Köhler et al. 1999; Maddocks and Oyston 2008). This efflux pump is related to the increased resistance of chloramphenicol, trimethoprim and fluoroquinolones (Köhler et al. 1997a, b). As a global transcriptional regulator, in addition to regulation of the *mexEF*-*oprN* operon and a neighboring gene *mexS* (Köhler et al. 1999), MexT also regulates the expressions of more than 40 genes (Tian et al. 2009a). Because MexEF-OprN pump exports the PQS precursor HHQ (Lamarche and Déziel 2011), MexT regulates many QS-controlled phenotypes. Inactivation of MexEF in the background of the LasR mutant also elevates the QS-controlled pyocyanide production (Kostylev et al. 2019). On the other hand, overexpression of MexT leads to the attenuated QS-controlled phenotypes, such as the production of pyocynin, C4-HSL, cyanide, elastase and rhamnolipids (Köhler et al. 2001; Tian et al. 2009b).

Mutations in the *mexT* gene have been reported in clinical isolates from CF patients (Smith et al. 2006), intestinal tissues (Gilbert et al. 2012), and commonly used laboratory PAO1 strains (Luong et al. 2014; Maseda et al. 2000). Inactivation of MexT in those strains exhibited increased pyocyanin production, high swarming motility, reduced chloramphenicol resistance and increased destructive capability on tissues (Gilbert et al. 2012; Luong et al. 2014). Mutations in *mexT* gene were also found in the PAO1 LasR mutant grown in protein-based broth (Oshri et al. 2018), and supplementation of synthetic C4-HSL or co-culture with clinical isolates secreting C4-HSL can greatly accelerate this genetic adaptation (Kostylev et al. 2019).

The *P. aeruginosa* strain PAO1, obtained from a wound in Melbourne, Australia (Holloway and Morgan 1986; Holloway 1955), serves as a reference strain commonly used for *Pseudomonas* research in laboratories worldwide. Laboratory-propagated and -maintained PAO1 strains have been found to contain genome variations (Chandler et al. 2019; Klockgether et al. 2010), while QS characteristics in different laboratory PAO1 strains remain illustrative. To address this concern, we systematically surveyed QS profiles in three PAO1 strains derived from different sources. We used skim milk plate and QS reporter assays to screen QS phenotypes in laboratory PAO1 strains. Our study found that the PAO1-z strain showed reduced QS activity when compared to the other two laboratory PAO1 strains. Further genetic analysis revealed that the *lasR* gene in the PAO1-z strain was disrupted by a 3-bp insertion, resulting in a nonfunctional LasR protein and an impaired Las QS system. The WGS analysis further revealed the *mexT* gene, which encodes a global transcriptional regulator, has an 18-bp deletion mutation. Although the PAO1-z strain contains a nonfunctional LasR, the Rhl QS system in PAO1-z is activated by the MexT inactivation, producing elevated Rhl-regulated products such as pyocyanin, cyanide and elastase. Our findings revealed that QS adaptation occured in the laboratory PAO1 strain, which probably underwent a QS evolution scenario that an impaired Las QS system was followed by the activation of the Rhl QS system.

## Materials and methods

### Bacterial strains and growth

*Pseudomonas aeruginosa* PAO1-u strain was obtained from E. Peter Greenberg (University of Washington, USA), PAO1-m strain from Matthew Parsek (University of Washington, USA), and PAO1-z strain was collected from Zhang Lianhui (SCAU, China). The PAO1 strains and the mutant derivatives were grown in Luria Bertani (LB) broth containing 10 mg/ml tryptone, 5 mg/ml yeast extract, 10 mg/ml NaCl at 37 °C. Unless otherwise specified, *P. aeruginosa* strains were cultured in 14-mm FALCON tubes (Corning, USA) containing 3 mL medium, with shaking (225 RPM) at 37 °C. *Escherichia coli* was grown in LB broth at 37 °C. The bacterial strains used in this study are listed in Additional file 1: Table S3.

### Construction of *P. aeruginosa* mutants

Either point mutation or full gene knocking out was based on the homologous recombination exchange approach as described previously (Rietsch et al. 2005). Briefly, about 500 ~ 1000 bp of DNA flanking the targeted single nucleotide substitution or full length of gene of interest were PCR-amplified and cloned into pEXG-2 vector (Gentamycin resistance, Gm) (Rietsch et al. 2005; Stover et al. 2000) with the Vazyme ClonExpress II One Step Cloning kit (Vazyme Biotech, Nanjing, China), generating pEXG-flanking constructs. The primers used for cloning are listed in Additional file 1: Table S2. The pEXG-flanking construct was mobilized into *P. aeruginosa* strain by triparental mating with the help of *E. coli* PRK2013 strain (Kanamycin resistance, Km). Point mutation or full-length gene deletion mutants were first selected on Pseudomonas Isolation agar (PIA) containing Gm100 and further selected on LB agar containing 10% sucrose. All mutants were confirmed by PCR amplification and subsequent DNA Sanger sequencing.

### Constitutive expression of an extra copy of *mexT*

Open reading frame sequences of gene *mexT* were fused with 265 bp native promoter region sequence and cloned into pUC18T-mini-Tn7T-Gm (NCBI accession number: AY599232) (Choi and Schweizer 2006a), generating miniTn7-*mexT*. The miniTn7-*mexT* was integrated into the neutral site of genome of PAO1 strains together with the transformation of helper plasmid pTNS2 (NCBI accession number: AY884833). Integration event was confirmed by PCR amplification and DNA sequencing. The excision of Gm resistance was performed with pFLP2 plasmid (NCBI accession number: AF048702) (Choi and Schweizer 2006b) and selected on LB agar containing 5% sucrose.

### QS reporter assay

P*lasI*-GFP and P*rhlA*-GFP (Feltner et al. 2016) were used to quantify the LasR- and RhlR-responsive activities, respectively. P*lasI*-GFP and P*rhlA*-GFP were mobilized into PAO1 strains and selected on LB agar plate (Gm). PAO1 strains bearing QS reporter plasmids were grown in LB broth containing 50 mg/mL gentamycin for 12 h. Cell pellets were washed with PBS and subjected to microplate reader (Synergy H1MF, BioTek Instruments, USA) for GFP measurement.

### Skim milk assay

Total proteolytic activity of *P. aeruginosa* strains was evaluated through the skim milk assay, in which the tested strains form a zone of clearing on skim milk agar plate. Individual colonies were spotted on the skim milk agar plates (25% LB, 4% skim milk, 1.5% agar). The protease-catalyzed zones were photographed after incubation at 37 °C for 18 h.

### Pyocyanin production

Overnight cultures of *P. aeruginosa* grown in LB broth were diluted into 4 mL LP medium (20 g/L Gelation peptone, 1.4 g/L MgCl_2_, 10 g/L K_2_SO_4_, 10 mM Glycerol, pH 7.2) to reach a starting OD_600_≈0.02 and spotted on to the LP agar plate for visualization.

### Cyanide measurement

Cells grown in LB broth overnight were diluted to OD600≈0.1 and spotted on to the peptone agar plate (2% peptone in 1.5% agar). The plates were covered with filter paper soaking with 5 mg/mL Copper (II) ethylacetoacetate and 5 mg/mL 4,4'-methylenebis- (N,N-dimethylaniline) and incubated at 37 °C for 18 h.

### Elastase production assay

*P. aeruginosa* strains grown in LB broth overnight were diluted to OD600≈0.02 in 2 mL LB broth for shaking at 37 °C for 8 h. The cells were spin down at 16,000*g* × 2 min and 500 μl supernatants were transferred to a tube containing the same amount of ECR buffer (0.1 M Tris–Cl, 1 mM Cacl2, 5 mg/mL Congo red, pH 7.2) at 37 °C for 2 h. Reaction was stopped by adding 100 μl EDTA (0.12 nM). Cells were pelleted at 5000*g* × 5 min and supernatants were measured at OD495 nm.

### Quantitative real-time PCR (qRT-PCR)

Total RNA of PAO1 strains and derivatives were reverse transcribed using the HiScript II 1st Strand cDNA Synthesis Kit (Vazyme Biotech, Nanjing, China) following the manufacturer’s instructions. The obtained cDNA was used for qRT-PCR. Quantification reactions containing SYBR qPCR Master Mix (Vazyme Biotech, Nanjing China) were prepared in 96-well plates and run in StepOnePlus Real-Time PCR System (Applied Biosystems, USA) as recommended. Primers used for qRT-PCR are listed in Table S2. The expression of targets of interest was normalized to the expression level of the *proC* gene. Reactions were performed in triplicate.

### Whole-genome sequencing by Illumina HiSeq

1 μg of microbial genomic DNA was sonicated to an average size of ~ 350 bp by Covaris-S220 ultrasonicator (Covaris, Woburn, MA, USA). Illumina DNA fragment library preparation was performed following the manual of Next-Generation Sequencing DNA library preparation kit (Novagen). Briefly, the fragmentated DNA products were end repair and ligated with an adapter. Adapter-ligated products were purified using AMPure XP beads (Agencourt-Berkman Coulter, USA) and enriched through PCR amplification using the custom adapter-specific primers. The obtained unbiased short read library was further cleaned up with AMPure XP beads. Pair end Illumina HiSeq PE150 sequencing was performed with an Illumina Novaseq 6000 sequencing system.

### Analysis of Illumina HiSeq short reads

Raw short reads were subjected to quality control including removing adapters using cutadapt (v1.16) by Novagen (Novagen, China), yielding clean short reads. Clean short reads were mapped to the PAO1 reference genome (accession number NC_002516.2) with bwa (v0.7.15-r1140). The mapped short reads were subjected to a genome-wide genetic variant calling using Samtools (v1.5) and Breakdancer (v1.4.5) software. The statistics of short read analysis is listed in Additional file 1: Table S1.

### Used software

Following bioinformatics software were used in this study:

- Cutadapt, version 1.16 (https://github.com/marcelm/cutadapt/),
- Bwa, version 0.7.15-r1140 (http://bio-bwa.sourceforge.net) (Li and Durbin, 2009),
- Samtools, version 1.5 (http://samtools.sourceforge.net) (Li et al. 2009),
- Breakdancer, version v1.4.5 (https://github.com/genome/breakdancer) (Chen et al. 2009),
- Perl, version v5.22.1. (https://www.perl.org/),
- FastQC, version fastqc_v0.11.5 (https://www.bioinformatics.babraham.ac.uk/projects/fastqc/),
- Hisat2, version 2.1.0 (https://daehwankimlab.github.io/hisat2/).(Kim et al. 2015),
- HTSeq, version 0.11.1 (https://htseq.readthedocs.io/en/release_0.11.1/count.html). (Anders et al. 2015), and DESeq2, (Love et al. 2014).

### Statistical analysis

Statistical analyses were performed using Excel and R software (http://www.R-project.org/).

## Results

### QS-related phenotypes in *P. aeruginosa* laboratory strains.

**Fig. 1:**
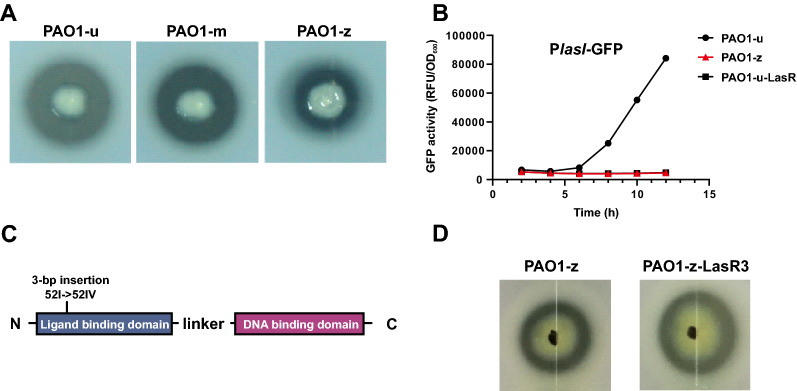
QS activity profiles in *P. aeruginosa* laboratory strains. **A** Protease activity is shown in skim milk assay of three *P. aeruginosa* laboratory strains. An equal amount of bacteria were spotted on to the skim milk plate and photographed after 18 h of incubation. **B** The LasR-responsive activity in PAO1-u strain, PAO1-z strain and PAO1-u-LasR mutant. LasR-responsive activity was reflected by the fluorescence level of the P*lasI*-GFP reporter. The expression level of GFP in the tested strain was measured by the microplate reader and reported as relative fluorescence units (RFU). **C** Illustration of 3-bp insertion in the ligand-binding domain of the *lasR* gene in the PAO1-z strain. **D** Protease activity of PAO1-z strain and PAO1-z-LasR3 mutant in skim milk plate. PAO1-z-LasR3 mutant, PAO1-z strain with deleting the *lasR3* variant

To investigate the QS characteristics in *P. aeruginosa* laboratory strains, we examined QS-related phenotypes of PAO1 strains commonly used in different labs. We tested three laboratory PAO1 strains, designated as PAO1-m, PAO1-u and PAO1-z. They were maintained in LB nutrition-rich broth, in which PAO1 generally is thought not to be subjected to selection pressures. The skim milk plate assay was used to assess their protease activity, which is mainly controlled by the Las QS system and partially by the Rhl QS system (Pearson et al. 1997). Although all of the strains showed protease-positive activity in the skim milk plate, PAO1-z displayed a smaller protease-catalyzed zoom than that in the PAO1-m or the PAO1-u strain (Fig. 1A). This reduced proteolytic activity of PAO1-z implied that its QS activity was compromised to some degree, presumably resulting from genetic mutations associated with QS-related genes.

We therefore sequenced the component genes of the Las and Rhl QS systems in the PAO1-z strain, and found that the *lasR* gene has a 3-bp insertion in the ligand-binding domain (LBD) (herin referred to as LasR3 variant), resulting in a disrupted open reading frame (Fig. 1C). Furthermore, the Las QS reporter result revealed that the fluorescence level of P*lasI*-GFP in PAO1-z was as low as the PAO1-u-LasR mutant (Fig. 1B). Therefore, the LasR3 variant protein in PAO1-z was completely inactive. In addition, when the full-length of *lasR*3 was deleted from PAO1-z, the resulting PAO1-z-LasR3 mutant still remained the similar proteolytic activity as the parent PAO1-z strain (Fig. 1D), suggesting LasR-3 variant in PAO1-z does not contribute to either the active proteolysis or the active QS system. Given that LasR is atop of the QS hierarchy (Lee and Zhang 2015), this finding raises the question how the production of QS-regulated protease was restored in the PAO1-z strain. Taken together, these findings show that different laboratory PAO1 strains have developed distinct QS phenotypes resulting from respective genome innovations. The Las QS activity in the PAO1-z strain was functionally disrupted due to a 3-bp insertion occuring in the *lasR* gene, and the retained QS-controlled proteolysis implies that the QS hierarchy was re-adapted in the PAO1-z strain.

### Inactivating MexT is responsible for rewired QS phenotypes in PAO1-z

To identify the genetic changes responsible for the altered QS phenotypes in PAO1-z, we performed the whole-genome short read re-sequencing (WGS) analysis. Short reads of PAO1-z were mapped to the reference PAO1 genome (NCBI accession number: NC_002516.2). Relative to the PAO1-u genome, PAO1-z has only a few genomic differences, including single-nucleotide substitutions, insertions and deletions. They include a 3-bp insertion in the *lasR* gene, an 18-bp deletion in the *mexT* gene, three single-nucleotide substitutions occurring in the *tsap* gene, *gtrS* gene and the noncoding region, respectively (Table 1). These DNA elements bearing mutations are thus the potential candidates responsible for the altered QS phenotypes in PAO1-z.

**Table 1:** Identification of genome mutations in the PAO1-z strain

Nucleotide change	Amino acid change	Mutation type		Encoding	Targeted gene		Locus tag	Product
A1558324→ATCG	52I→IV	3-bp insertion		CDS	*lasR*		PA1430	Transcriptional regulator
C22278→T	No change	SNP		CDS	*tsaP*		PA0020	T4P secretin-associated protein
C3582640→A	157P→Q	SNP		CDS	*gtrS*		PA3191	Glucose transport sensor
GCGCTGTCGCGCCTGCGCA2807724→G	Truncated protein	18-bp deletion		CDS	*mexT*		PA2492	Transcriptional regulator
C5036907- > G		SNP		Noncoding				

Whole-genome re-sequencing (WGS) was performed with the PAO1-z strain. Short reads were mapped to the reference PAO1 genome (NC_002516.2). Genome mutations were identified relative to the PAO1-u strain

**Fig. 2:**
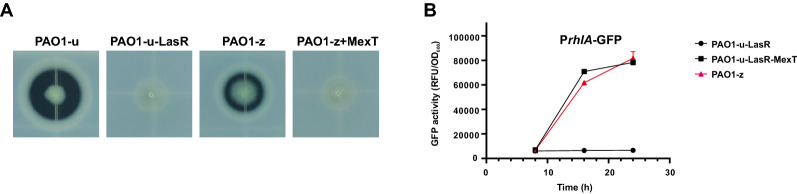
Inactivating MexT responsible for the active Rhl QS phenotypes in PAO1-z. **A** Skim milk plate assay of indicated strains. The same amounts of bacteria were spotted onto the skim milk plate and photographed after 18 h incubation. **B** Detection of fluorescence of P*rhlA*-GFP reporter in indicated strains. The expression level of GFP in the tested strain was measured by the microplate reader and reported as relative fluorescence units (RFU). PAO1-u-LasR, PAO1-u strain with deleting the full-length of the *lasR* gene; PAO1-z + MexT, PAO1-z strain was complemented with a construct expressing the *mexT* gene from the PAO1-u strain; PAO1-u-LasR-MexT, PAO1-u strain with deleting both of the *lasR* and *mexT* genes

Because either overexpression or deletion of the *mexT* gene has been shown to substantially influence Rhl QS activity (Köhler et al. 2001; Tian et al. 2009b), we hypothesized that the 18-bp-deleted *mexT* gene in PAO1-z may encode a nonfunctional protein. In the skim milk plate assay, a functional *mexT* copy from PAO1-u was transferred into PAO1-z, resulting in a protease-deficient phenotype in the recombinant strain (Fig. 2A). This complementation result indicated the 18-bp-deleted *mexT* gene in PAO1-z was functionally inactive. Furthermore, this copy of *mexT* gene from PAO1-z was mobilized into PAO1-u-LasR-MexT double mutant but did not affect the protease activity in the resultant strain (Additional file 1: Figure S1). All these results indicated that the *mexT* gene in PAO1-z encodes a nonfunctional protein. Similar to the LasR-MexT mutant, PAO1-z also showed increased Rhl QS activity, as reflected by the P*rhlA*-GFP reporter (Fig. 2B) and elevated productions of pyocyanin pigment, cyanide and elastase when compared to the LasR mutant (Additional file 1: Figure S2). Shown by selected QS genes, the qRT-PCR analysis also confirmed the elevated Rhl QS activity as well as the PQS QS activity in the PAO1-z strain (Additional file 1: Figure S3). Therefore, we concluded that PAO1-z has an inactive Las QS system, but the Rhl QS system is restored by the *mexT* mutation.

We did not further investigate the roles of the *tsap* gene and the *gtrS* gene for the QS adaptation in PAO1-z. The single-nucleotide substitution in the *tsap* gene did not affect the coding amino acids (Table 1). Meanwhile, the *gtrS* genes encode a glucose transport sensor, which is less likely to be involved in the QS pathway regulation. We therefore reasoned that the *lasR* and *mexT* genes are most likely responsible for the evolution of QS adaptation in PAO1-z. Our study suggests that under certain laboratory conditions, the mutation that occurred in the *lasR* gene resulted in the inactivation of the LasR QS system, followed by the adaptive mutation in the *mexT* gene leading to the activation of the Rhl QS system.

## Discussion

In our study, the adaptive mutations in the *lasR* and the *mexT* gene, which are responsible for the changes of the QS hierarchy, were identified in a commonly used laboratory PAO1-z strain cultured in nutrition-rich conditions. We assume that the *lasR* mutations inactivated QS first, followed by a secondary *mexT* mutation that reprogramed the QS hierarchy in the PAO1-z strain. It is very unlikely an opposite scenario of mutation occurred. *P. aeruginosa* has complex regulatory networks (Huang et al. 2019; Lee and Zhang 2015) that allow this bacterium to rearrange its QS hierarchy, and our findings demonstrate that *P. aeruginosa* could utilize QS to efficiently adapt to environments. QS homeostasis has previously been observed in LasR mutant cells during the stationary phase or under the starvation condition. In the late stationary phase, Rhl and PQS QS systems could achieve to be self-active in the LasR-null mutant (Dekimpe and Deziel 2009; Diggle et al. 2003), while LasR mutant cultured in protein-based broth could also emerge *mexT* mutations to activate the LasR-independent Rhl and PQS QS systems (Kostylev et al. 2019; Oshri et al. 2018). In comparison, our study found that the PAO1-z strain maintained in nutrient-rich broth also underwent genome variations that resulted in QS hierarchy autoregulation. Future research will be needed to fully elucidate the specific condition that could induce the QS autoregulation in the PAO1-z strain.

With adaption to the deteriorating environments in CF lungs, *P. aeruginosa* isolates in chronic infection appear evolutionary changes, resulting in a wide spectrum of colony variants that are hypermutable, nonmotile, nonflagellated, liposaccharide-deficient, resistant to antibiotics, auxotrophic (Oliver et al. 2000; Winstanley et al. 2016). Owing to high genetic and phenotypic diversities, genetic knowledge in clinical isolates is difficult to be translated into the understanding of the corresponding adapted phenotypes. In *P. aeruginosa* CF chronical infection isolates, mutations in the QS master regulator *lasR* gene were commonly identified (D'Argenio et al. 2007; Hoffman et al. 2009; Smith et al. 2006). Although the *lasR* mutation usually yields a nonfunctional protein causing the paralysis of the QS system (Feltner et al. 2016), LasR-null isolates were capable of engaging in QS activity with producing QS-associated factors and QS signal molecules (Bjarnsholt et al. 2010; Chen et al. 2019; Cruz et al. 2020; Feltner et al. 2016). Although the microevolution has been reported across laboratory PAO1 strains worldwide (Chandler et al. 2019; Klockgether et al. 2010), the frequency of mutation in laboratory PAO1 strains was rather lower than that in clinical isolates. This observation was supported in our WGS data with the identification of only a few adaptative mutations in the long-term laboratory-maintained PAO1-z strain (Table 1). Meanwhile, our findings also demonstrate that the laboratory-adapted strain experienced QS adaptation. Therefore, by examining the respective genotype–phenotype association, the laboratory PAO1 strain could serve as a useful platform for studying *Pseudomonas* QS adaptation by subjecting the bacterium to conditions similar to those found in clinical settings.

Our study found that the laboratory strain PAO1-z underwent genome innovations that led to the alteration of QS hierarchy. In such situation, the QS characteristics of a laboratory PAO1 strain may probably have been masked to some extent. This will impact on and mislead the following QS-related researches if using the QS-altered PAO1 strains. Our findings suggest that as a first step toward a proper illustration of QS adaptability in PAO1 strains, it is crucial to verify the potential effects derived from their genome variations.

## Supplementary Information

**Additional file 1.** Supplementary information in this study.

## Data Availability

The short-read sequencing data set have been deposited in the Sequence Read Archive (SRA) (www.ncbi.nlm.nih.gov/sra) under accession number PRJNA779485.

## Code availability

Used software were listed in the section of Materials and methods.
