# Supplementary material for: Spontaneous quorum-sensing hierarchy reprogramming in Pseudomonas aeruginosa laboratory strain PAO1
Source: AMB Express. 2022 Jan 26;12:6. doi: 10.1186/s13568-022-01344-7 (PMC8792115; doi:10.1186/s13568-022-01344-7)
Supplement: Supplementary file 1 — Additional file 1. Supplementary information in this study. [file 13568_2022_1344_MOESM1_ESM.doc]

**AMB Express**

Supplementary Information for:

**Spontaneous quorum-sensing hierarchy reprogramming in *Pseudomonas aeruginosa* laboratory strain PAO1**

Xiaoyan Cheng^1,2*^, Mingqi Lu^1,2*^, Huifang Qiu^1,2^, Yuanhao Li^1,2^, Linfeng Huang^3,4^, Weijun Dai^1,2^

^1^Guangdong Laboratory for Lingnan Modern Agriculture, South China Agricultural University, Guangzhou, Guangzhou, 510642, China

^2^Guangdong Province Key Laboratory of Microbial Signals and Disease Control, Integrative Microbiology Research Center, South China Agricultural University, Guangzhou, 510642, China

^3^Department of Biomedical Sciences, City University of Hong Kong, Kowloon, Hong Kong SAR, China

^4^Division of Natural and Applied Sciences, Duke Kunshan University, Kunshan, Jiangsu, China

*These authors contribute equally

Send correspondence to: Weijun Dai

Integrative Microbiology Research Center, South China of Agricultural University, Guangzhou, 510642, China

Phone (office): +86-20-87575650

E-mail: daiweijun@scau.edu.cn

**Figure S1** Nonfunctioning MexT in PAO1-z.

**Figure S2 QS-controlled products** in PAO1-z.

**Figure S3 qRT-PCR analysis of QS-related and virulence genes in PAO1-z.**

**Table S1.** Reads mapping statistics of whole -genome re-sequencing data

**Table S2.** Oligonucleotides used in this study

**Table S3.** Bacterial strains used in this study

**
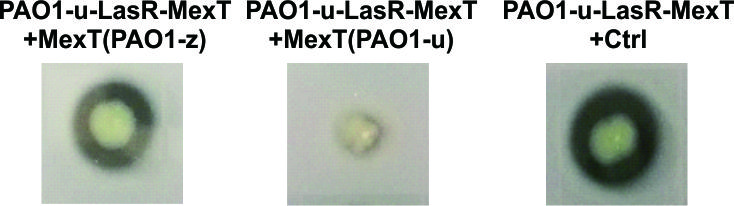
**

**Fig. S1 Nonfunctioning MexT in PAO1-z.**

Skim milk plate experiment of PAO1-u derivative strains. PAO1-u-LasR-MexT double mutant was complemented with the *mexT* gene from the PAO1-z strain or the PAO1-u strain. The *mexT* gene of PAO1-z or PAO1-u was cloned into a miniTn7 vector and transferred into PAO1-u-LasR-MexT double mutant. Colonies in skim milk plate were photographed after 18h incubation. PAO1-u-LasR-MexT+MexT(PAO1-z), PAO1-u-LasR-MexT mutant carrying the *mexT* gene from the PAO1-z strain; PAO1-u-LasR-MexT+MexT(PAO1-u), PAO1-u-LasR-MexT mutant carrying the *mexT* gene from the PAO1-u strain; PAO1-u-LasR-MexT+Ctrl, PAO1-u-LasR-MexT mutant carrying a miniTn7 empty vector.


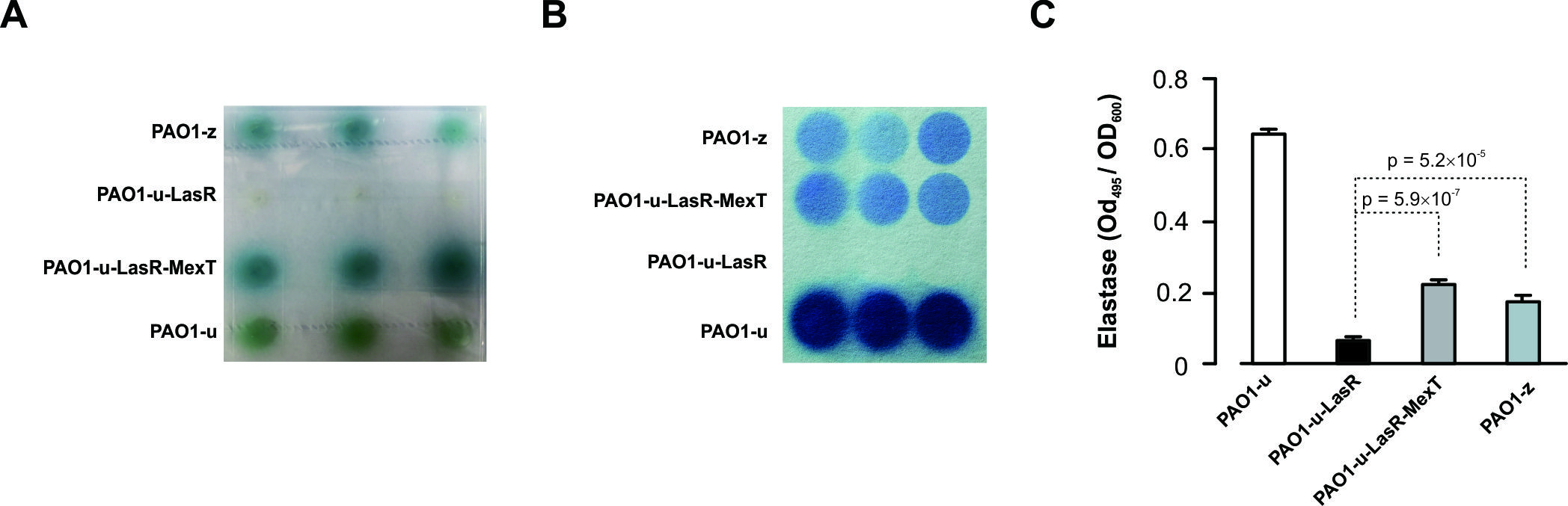


**Fig. S2 QS-controlled products in PAO1-z.**

(A) Pyocyanin production in the indicated strains. Indicated strains were spotted on the low phosphate (LP) agar plate and photographed after post inoculation 18h. (B) Cyanide levels of colonies. Cyanide-sensitive filter paper were photographed after post bacterial inoculation 18h. (C) Elastase quantification of cultures. Elastase production were quantified at OD495 divided by OD600 of each assayed strain, respectively LasR mutant and LasR-MexT double mutant are derived from the PAO1-u strain background. Data are presented as mean ± SD. n =3 biologically independent samples, *t*-test.

**
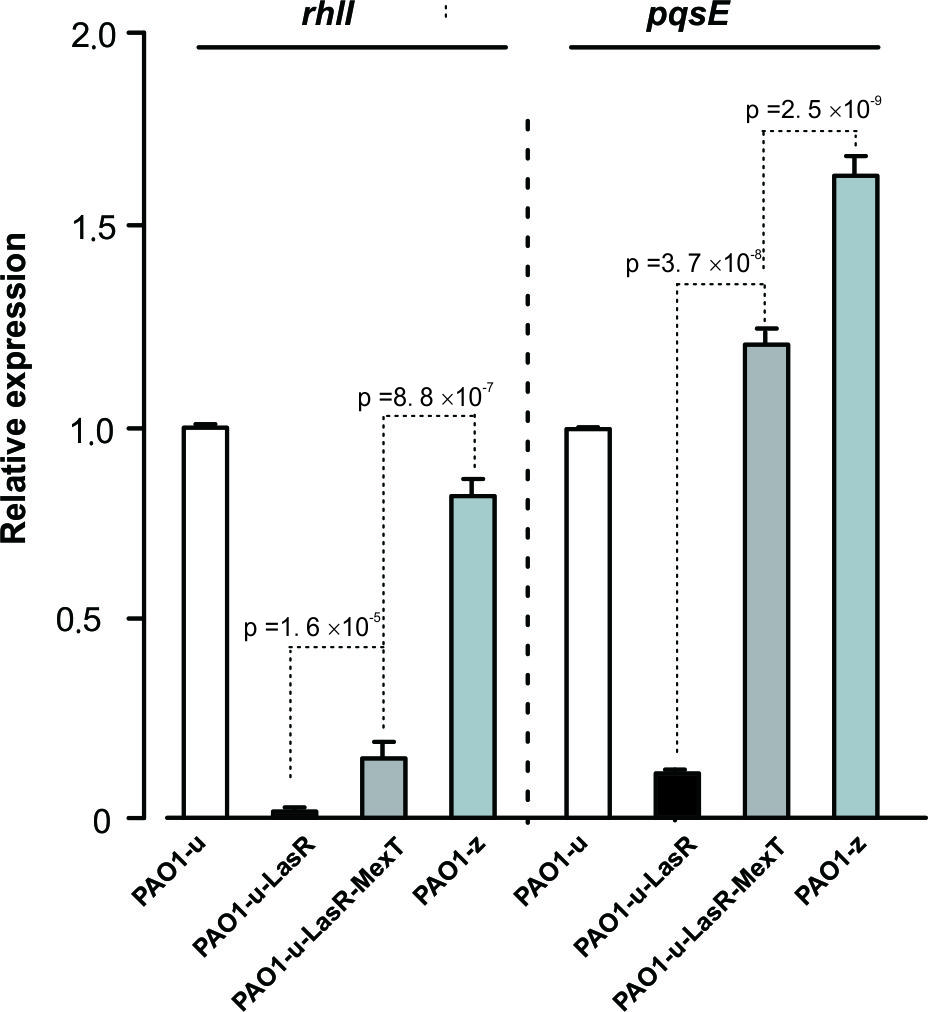
**

**Fig. S3 qRT-PCR analysis of QS-related and virulence genes in PAO1-z.**

qRT-PCR was used to evaluate indicated genes of designated strains. Relative expression was normalized to the *proC* gene. Used primers are shown in Table S2. Data are means ± SD (3 independent RNA extractions; n = 3). P-values were calculated using *t*-tests.

**Table S1.** Reads mapping statistics of whole -genome re-sequencing data

| **Strain** | **No. of total reads** | **No. of mapped reads** | **Percentage of mapped reads (%)** | **Genome coverage (%)** | **Sequencing depth** |
| --- | --- | --- | --- | --- | --- |
| PAO1-z | 15,194,739 | 11,602,267 | 76.36% | 99.98% | 699 × |
| Shor reads were mapped using the reference genome of PAO1 strain (NC_002516.2). | | | | |  |

**Table S2.** Oligonucleotides used in this study

| **ID** | **Name** | **Sequence, 5' to 3'** |
| --- | --- | --- |
| **Detection of peXG-2 based constucts** | | |
| 15 | pGEX2-F | GAAATGCCGTATGTTTCCTT |
| 16 | pGEX2-R | TCCGCGTTTCCAGACTTTAC |
| **PCR amplification of *lasR* gene** | | |
| 38 | *lasR*-F | GTGGGCTGACTGGACATCTT |
| 39 | *lasR*-R | TCAGAGCAATGGCTTCACAC |
| **Deletion of *lasR* gene** | | |
| 126 | *lasr*3-F1 | ggccgacagtgaacccgg |
| 127 | *lasr*3-R1 | accgaacttccgccgaatat |
| 128 | *lasr*3-F2 | GACTCTAGAGGATCCCCcccggccgacagtgaacccgg |
| 129 | *lasr*3-R2 | CCGAATTCGAGCTCGAGCCCaccgaacttccgccgaatat |
| **Deletion of *mexT* gene** | | |
| 55 | *mexT*-A-F1 | GACATTGACGCCCTTGCCCTC |
| 56 | *mexT*-A-R1 | AGCCATTATCAATAACGACGGGTG |
| 57 | *mexT*-A-F2 | CATAAATGTAAAGCAAGCTTCTGCAGGTGACATTGACGCCCTTGCCCTC |
| 58 | *mexT*--B-F1 | GCCCTCCGGCAGCTACCCGCACGA |
| 59 | *mexT-*-B-R1 | CTGGGCTTCGTTGACGCTCC |
| 60 | *mexT*--B-F2 | CCACCCGTCGTTATTGATAATGGCTGCCCTCCGGCAGCTACCCGCACGA |
| 61 | *mexT*--B-R2 | ATTAAGGTACCGAATTCGAGCTCGAGCCCCTGGGCTTCGTTGACGCTCC |
| **Expression of miniTn7-based *mexT*** | | |
| 230 | *mexT*-245bp-F1 | TGACGCCAGAGCACATCCTTCCA |
| 231 | *mexT*-238bp-R1 | TGTTCCATGCTTGACTCCGCCAGT |
| 232 | miniTn7-*mexT*-245bp-F2 | catgcatgagctcactagtgTGTTCCATGCTTGACTCCGCC |
| 233 | miniTn7-*mexT*-238bp-R2 | ttcgcgaggtaccgggcccaTGACGCCAGAGCACATCCTT |
| **pPROBE-GT containing *lasI* promoter or *rhlA* promoter or *pqsA* promoter** | | |
| 253 | P*rhlA*-GFP-*HindIII*-F | TTTTTAAGCTTGCATGCGAGGCCTGCGAA |
| 254 | P*rhlA*-GFP-*BamHI*-R | AAAAAAGGATCCTCTAGAAACCGATACCAACAGACTTTCGC |
| 255 | P*lasI*-GFP-*HindIII*-F | GGAATTGGGGATCGGAAGCTTTGCTCTGATCTTTTCGGACG |
| 256 | P*lasI*-GFP-*EcoRI*-R | CTTTAGTTAGTTAGGGAATTCCTTCACTTCCTCCAAATAGGA |
| 259 | PROBE detect F1 | GTTTTCCCAGTCACGACGTT |
| 260 | PROBE detect R1 | GTTGGCCATGGAACAGGTAG |
| **qRT-PCR primer** | | |
| 0289 | *proC*_q-F1 | CGTCGTGGTCCTGTCGGTCA |
| 0290 | *proC*_q-R1 | GGCGGCGATGGAGACGATCA |
| 0313 | *rhlI*_q-F1 | TACCGGCATCAGGTCTTCA |
| 0314 | *rhlI*_q-R1 | GTTTGCGGATGGTCGAACT |
| 0317 | *pqsE*_q-F1 | TCAACTGGATGATGACCTGTG |
| 0318 | *pqsE*_q-R1 | GGCTGATCCCTCCTTCAAC |

**Table S3.** Bacterial strains used in this study

| **Bacterial strain** | **Description** | **Reference or source** |
| --- | --- | --- |
| ***P. aeruginosa*** |  |  |
| PAO1-u | Wild-type PAO1 strain | E. Peter Greenberg |
| PAO1-m | Wild-type PAO1 strain | Matthew Parsek |
| PAO1-z | Wild-type PAO1 strain | Zhang Lianhui |
| PAO1u--LasR | PAO1-u strain containing an unmarked, in-frame *lasR* deletion | ref (1) |
| PAO1-u-LasR-MexT | PAO1-u- LasR mutant with MexT deletion | This study |
| PAO1-z-LasR3 | PAO1-z strain with LasR3 deletion | This study |
| PAO1-u-LasR-MexT+MexT (PAO1-u) | PAO1-u-LasR-MexT strain complemented with MexT from PAO1-u | This study |
| PAO1-u-LasR-MexT (PAO1-z) | PAO1-u-LasR-MexT strain complemented with MexT from PAO1-z | This study |
| PAO1-u-LasR-MexT(Ctrl) | PAO1-u-LasR-MexT strain carrying a empty pUC18T-miniTn7T plasmid | This study |
| ***E. coli*** |  |  |
| DH5α | *supE44 lacU169( 80lacZ M15) hsdR17 recA1 endA1 gyrA96 thi-1 relA1 pir* | Laboratory collection |
| ***plasmid*** |  |  |
| pEXG2 | Allelic exchange vector with pBR origin, gentamicin reistance, sacB,Gm^r^ | ref (2) |
| PRK2013 | Helper plasmid; ColE1 replicon,Mob^+^ Tra^+^ Km^r^ | ref (3) |
| pUC18T-miniTn7T | Broad host-range vectors used for integration of single-copy genes into bacterial chromosomes at a neutral site | ref (4) |
| pTNS2 | Helper plasmid, Gm^r^ | ref (5) |
| pFLP2 | Source of FLP recombinase, sacB, oriT, rep, Ap^r^ | ref (6) |
| P*lasI*-GFP | pPROBE-GT vector containing *lasI* promoter fragment, Gm^r^ | ref (7) |
| P*rhlA*-GFP | pPROBE-GT vector containing *rhlA* promoter fragment, Gm^r^ | ref (7) |
| 1. Wang, M., Schaefer, A.L., Dandekar, A.A., and Greenberg, E.P. (2015). Quorum sensing and policing of *Pseudomonas aeruginosa* social cheaters. Proceedings of the National Academy of Sciences 112, 2187-2191. | | |
| 2. Rietsch, A., Vallet-Gely, I., Dove, S.L., and Mekalanos, J.J. (2005). ExsE, a secreted regulator of type III secretion genes in *Pseudomonas aeruginosa*. Proceedings of the National Academy of Sciences 102, 8006-8011 | | |
| 3. Figurski, D.H., and Helinski, D.R. (1979). Replication of an origin-containing derivative of plasmid RK2 dependent on a plasmid function provided in trans. Proceedings of the National Academy of Sciences 76, 1648-1652. | | |
| 4. Choi, K.-H., and Schweizer, H.P. (2006a). mini-Tn7 insertion in bacteria with single attTn7 sites: example *Pseudomonas aeruginosa*. Nature protocols 1, 153. | | |
| 5. Choi, K.-H., Gaynor, J.B., White, K.G., Lopez, C., Bosio, C.M., Karkhoff-Schweizer, R.R., and Schweizer, H.P. (2005). A Tn 7-based broad-range bacterial cloning and expression system. Nature methods 2, 443-448. | | |
| 6. Choi, K.-H., and Schweizer, H.P. (2006b). mini-Tn 7 insertion in bacteria with single att Tn 7 sites: example *Pseudomonas aeruginosa*. Nature protocols 1, 153. | | |
| 7. Feltner, J.B., Wolter, D.J., Pope, C.E., Groleau, M.-C., Smalley, N.E., Greenberg, E.P., Mayer-Hamblett, N., Burns, J., Déziel, E., and Hoffman, L.R. (2016). LasR variant cystic fibrosis isolates reveal an adaptable quorum-sensing hierarchy in *Pseudomonas aeruginosa*. MBio 7, e01513-01516. | | |
